# Microarray Probe Expression Measures, Data Normalization and Statistical Validation

**DOI:** 10.1002/cfg.312

**Published:** 2003-07

**Authors:** Silvia Saviozzi, Raffaele A. Calogero

**Affiliations:** Department of Biological and Clinical Sciences University of Torino c/o Az. Ospedaliera S. Luigi Regione Gonzole 10, Orbassano (TO) 10043 Italy

## Abstract

DNA microarray technology is a high-throughput method for gaining information on gene function. Microarray technology is based on deposition/synthesis, in an ordered manner, on a solid surface, of thousands of EST sequences/genes/oligonucleotides. Due to the high number of generated datapoints, computational tools are essential in microarray data analysis and mining to grasp knowledge from experimental results. In this review, we will focus on some of the methodologies actually available to define gene expression intensity measures, microarray data normalization, and statistical validation of differential expression.


**Comparative and Functional Genomics**
*Comp Funct Genom* 2003; **4**: 442–446.
Published online in Wiley InterScience (www.interscience.wiley.com). **DOI:** 10.1002/cfg.312

Conference Review

### Microarray probe expression measures, data normalization and statistical validation

Silvia Saviozzi and Raffaele A. Calogero*
*Department of Biological and Clinical Sciences, University of Torino, c/o Az. Ospedaliera S. Luigi, Regione Gonzole 10, Orbassano (TO) 10043, Italy*

*Correspondence to: Raffaele A. Calogero, Dipartimento di Scienze Cliniche e Biologiche, Università di Torino, Az. Ospedaliera S. Luigi, Regione Gonzole 10, Orbassano (TO) 10043, Italy. E-mail: raffaele.calogero@unito.it*

Received: 7 May 2003
Revised: 5 June 2003
Accepted: 6 June 2003

### Abstract

**DNA microarray technology is a high-throughput method for gaining information on gene function. Microarray technology is based on deposition/synthesis, in an ordered manner, on a solid surface, of thousands of EST sequences/genes/oligonucleotides. Due to the high number of generated datapoints, computational tools are essential in microarray data analysis and mining to grasp knowledge from experimental results. In this review, we will focus on some of the methodologies actually available to define gene expression intensity measures, microarray data normalization, and statistical validation of differential expression. Copyright © 2003 John Wiley & Sons, Ltd.**

**Keywords:** **microarrays; transcriptional profiling; probe set; differential expression**

### Introduction

Microarrays can be a valuable tool for defining transcriptional signatures bound to a pathological condition, or to rule out molecular mechanisms tightly bound to transcription. However, because our current knowledge of gene function in higher eukaryotes is still limited, microarray analysis frequently does not imply a final answer to a biological problem, but allows the discovery of new research paths that allow us to explore it from a different perspective. Additionally, it is essential to point out that a gold standard methodology to identify, with high sensitivity and precision, 'biologically meaningful' differential expression of genes is not yet available. Therefore, it is important to explore data by multiple approaches in order to generate a robust set of results [14].

Microarray technology was initially developed by Schena and co-workers [15] and it is based on spotting, in an ordered manner, on a solid surface, of thousands of EST sequences/genes. Microarrays have also been developed using photolithographic oligonucleotide synthesis (Affymetrix, Santa Clara, CA). cDNA spotted arrays are characterized by the use of one long stretch of bases for each gene, whereas in Affymetrix GeneChips up to 20 short oligonucleotides (probe set) are used to probe each gene/EST. Although Affymetrix arrays are far from being the ultimate solution for the characterization of gene expression they are, so far, one of the most used commercially available platforms for genome-wide transcriptional profiling analyses.

In this review, we will focus on computational approaches for GeneChip™ expression measures, data normalization and statistical validation.

### The Affymetrix GeneChip™

To assess the target hybridization specificity of each oligo (PM: perfect match) of the probe set, a 'negative control' oligonucleotide (MM: mismatch) is associated to each PM. This oligonucleotide has a sequence equal to PM but with a single central mismatch, which strongly destabilizes the hybridization of the target; the couple PM/MM is called a probe pair (the number, $j$, of probe pairs in a probe set ranges between 12 to 20). Consequently, evaluation of the hybridization signals on PM and MM

Copyright © 2003 John Wiley & Sons, Ltd.

probes gives an indication of the aptitude of any PM to identify a specific target, as a strong signal in the MM probe is a warning of the presence of cross-hybridizing targets. Target hybridization to Affymetrix GeneChips™ allows the generation of absolute intensity values describing the mRNA expression level. Therefore, to generate a 'virtual two-dye' experiment, two GeneChips™ have to be used.

### Probe set intensity signal calculation

To define a measure of expression representing the amount of the corresponding mRNA species it is necessary to summarize probe intensities for each probe set. Several model-based approaches to this problem have been proposed: the model-based expression index (MBEI [10]), the MAS 5.0 statistical algorithm from Affymetrix [1] and the robust multi chip average (RMA [7]).

Affymetrix MAS software [1] computes the probe set intensity signal as the anti-log of a robust average (Turkey biweight) of the values $\log(\mathrm{PM}_{ij} - \mathrm{CT}_{ij})$. CT is defined as a quantity equal to MM when MM < PM, but adjusted to be less than PM when MM ≥ PM, which is a quite frequent event [8]. A model for MAS 5.0 probe set intensity measures is $\log(\mathrm{PM}_{ij} - \mathrm{CT}_{ij}) = \log(\theta_i) + \varepsilon_{ij}, j = 1, \ldots, \mathrm{J}$. The expression quantity on array $i$ is represented by $\theta_i$ and $\varepsilon_{ij}$ is the error term which is equal to the variance for $j = 1, \ldots, \mathrm{J}$. Furthermore, MAS 5.0 assigns to each probe set an expression call (i.e. call P, gene is expressed; call A, gene is not expressed; call M, gene is marginally expressed).

The dCHIP software [10] computes the probe set intensity signal using a multiplicative model: $\mathrm{PM}_{ij} - \mathrm{MM}_{ij} = \theta_i\phi_j + \varepsilon_{ij}, i = 1, \ldots, \mathrm{I}, j = 1, \ldots, \mathrm{J}$. This model is based on the observation that the variation of a specific probe across multiple arrays could be considerably smaller than the variance across probes within a probe set [11], which indicates a strong probe affinity effect ($\phi_j$). $\phi_j$ can be calculated by dCHIP if a sufficient number of arrays (8–10) are available for the analysis. Fitting the model 'dCHIP expression measures' are obtained for each probe set. Furthermore, dCHIP allows the assessment of a standard error (SE) for each probe set intensity measure, which is an indicator of the hybridization quality to the probe set. SEs are useful for discarding probe sets with low hybridization quality.

The RMA expression measure (log scale Robust Multi-array Analysis), implemented in Affymetrix Oligonucleotide Array (Affy) R package [9], uses a model: $\mathrm{T}(\mathrm{PM}_{ij}) = \mathrm{e}_i + \mathrm{a}_j + \varepsilon_{ij}, i = 1, \ldots, \mathrm{I}, j = 1, \ldots, \mathrm{J}$, where T is the transformation that background corrects, normalizes, and logs the PM intensities, $\mathrm{e}_i$ is the $\log_2$ scale expression value found on arrays $i = 1, \ldots, \mathrm{I}$ and $\mathrm{a}_j$ is the log scale affinity effects for probes $j = 1, \ldots, \mathrm{J}$. According to Irizarry *et al.* [7], RMA has a better precision than MAS and dCHIP, especially for low expression values. Concerning the amount of true positives identified using spiked-in experiments, RMA performs slightly better than dCHIP, but much better than MAS [7]. In our hands, dCHIP compresses intensity signals with respect to MAS 5.0 measures in the low expression values (Figure 1A). Instead, RMA and MAS 5.0 detect intensity signals in a similar manner, even in the low expression values (Figure 1B). On the basis of published data and our observations, RMA seems the best approach, at present, to measure probe set expression levels, as it shows better sensitivity and specificity with respect to dCHIP and MAS.

### Data normalization

Array experimental conditions can strongly affect microarray hybridization intensities. It is assumed that sources of error are multiplicative and strongly affect true expression levels [6], especially if the genes are moderately expressed [13]. Therefore, normalization of gene expression data is a crucial preprocessing procedure that is essential for nearly all gene expression studies in which data from one array must be compared to data on an other array. A number of normalization approaches may be taken into account [5,12], however, a gold standard method for microarray data normalization has not been defined. Thus, the chosen method should be motivated by the application at hand and the goals of the data analysis.

MAS 5.0 performs a background correction across the entire array and also offers the possibility of performing data scaling, which is a mathematical technique that can minimize discrepancies due to variables such as sample preparation, hybridization

Copyright © 2003 John Wiley & Sons, Ltd. 

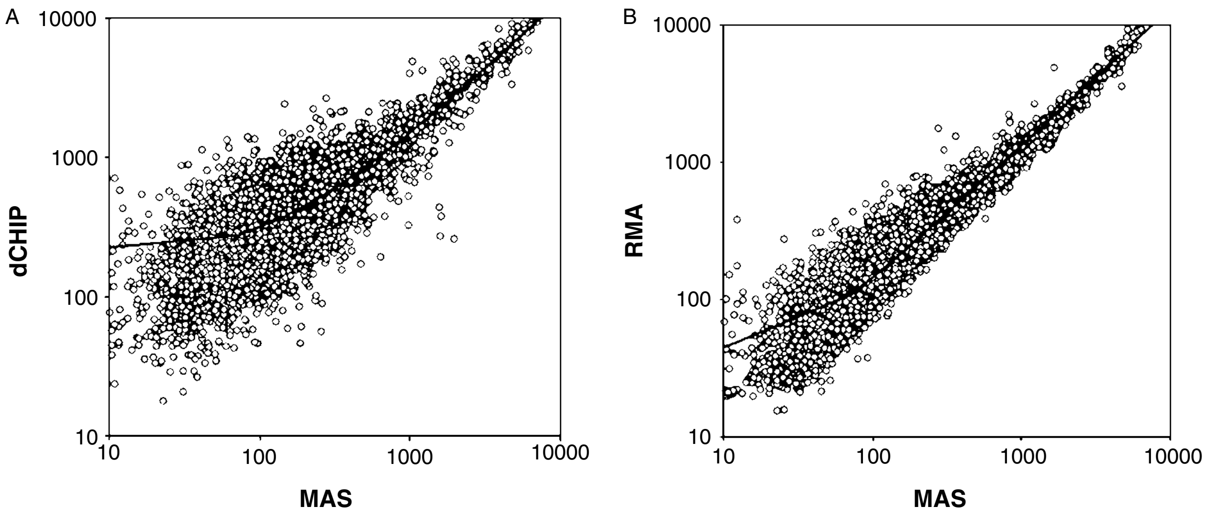

**Figure 1.** Comparison between expression measures performed on the same data set (MGU74Av2) using MAS 5.0 (scaling normalization), dCHIP 1.3 (invariant set normalization), and RMA (quantile normalization). (A) Expression measures obtained with dCHIP are plotted vs. MAS. (B) Expression measures obtained with RMA are plotted vs. MAS

conditions, staining or probe array lot. The scaling procedure does not affect the global similarity between the samples (Figure 2A, B; $r^2 = 0.9331$ for raw and scaled data).

The Invariant Set Normalization method is used in dCHIP [10] to normalize arrays. In this normalization procedure, an array with median overall intensity is chosen as the baseline array against which other arrays are normalized at probe intensity level. Subsequently, a subset of PM probes, with small within-subset rank difference in the two arrays, serve as the basis for fitting a normalization curve. This normalization method produces a better fitting of the replicates with respect to the MAS scaling procedure (Figure 2C; $r^2 = 0.9578$).

The Affy R package implements three different normalization procedures [3]: cyclic Loess, contrast-based method and quantile normalization (Figure 2D; $r^2 = 0.9540$). According to Bolstad [3], all the three methods reduce the variation of a probe set measure across a set of arrays to a greater extent than does the MAS 5.0 scaling method, and the quantile method performs better in terms of speed. The quantile method tries to make the same the distribution of probe intensities for each array in a set of arrays. The method is bound to the idea that a quantile–quantile plot shows that the distribution of two data vectors is the same if the plot is a straight diagonal. Since this concept can be extended to $n$ dimensions, it is possible to make a set of data have the same distribution if the points of the $n$ dimensional quantile plot are projected onto the diagonal [3]. This projection implies that it is possible to give the same distribution to each array by taking the mean quantile and substituting it as the value of the data item in the original data set.

As shown by the $r^2$ correlation coefficient in Figure 2, dCHIP gives better correlation between two replicates than the RMA/quantile normalization. Both dCHIP and RMA/quantile normalization perform better than MAS 5.0 scaling.

### Filtering

In microarray analysis, the exclusion from the dataset of non-informative probe sets, before getting to the statistical validation of the differential expression, is another step of the analysis. This step can be achieved by performing various filtering procedures [14]. The stringency of the filtering procedure could strongly affect (in a positive or a negative manner) the final results, as it can cause the loss of differentially expressed genes or increase the number of false positives contaminating the final results. In our lab, we remove from the original data set all probe sets which show, within all arrays, a signal very near to the background, using the MAS 5.0 absent calls (call A) [14]. Furthermore, we remove all probe sets showing low

Copyright © 2003 John Wiley & Sons, Ltd.

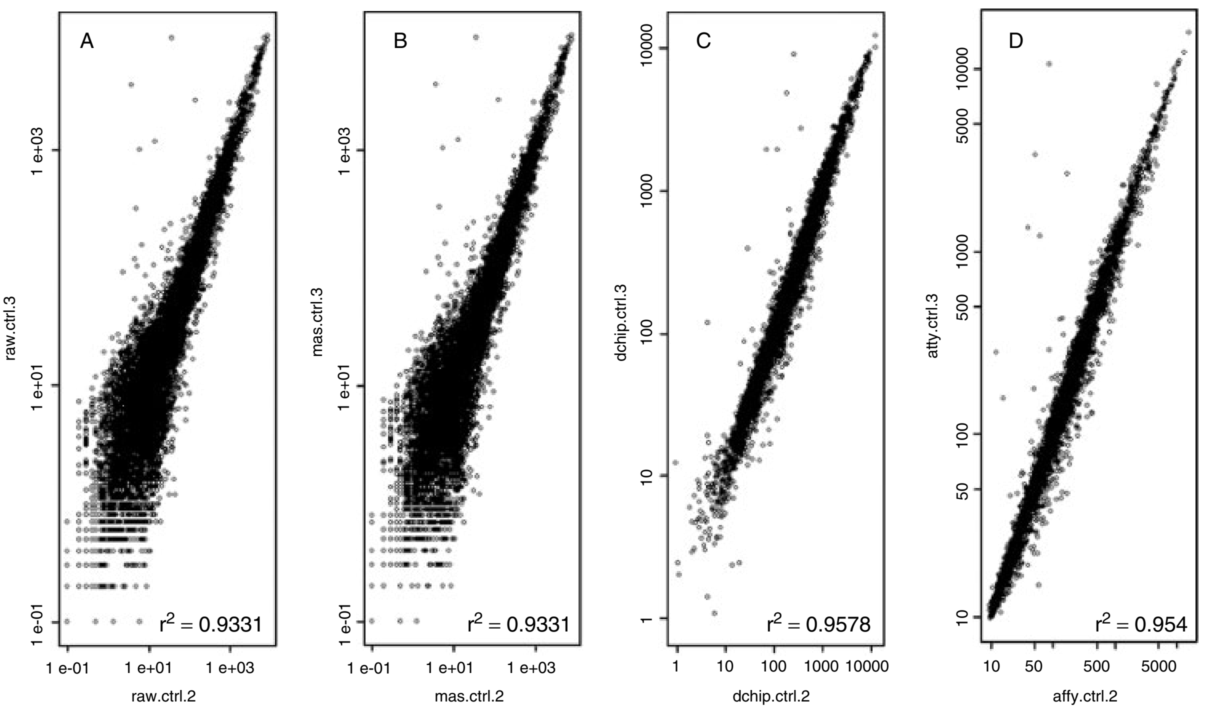

**Figure 2.** Comparison between various normalization methodologies. (A) Expression measures for two mouse breast biological replicates (MGU74Av2) are plotted against each other, as raw data. (B) As (A) but scaled according to MAS 5.0. (C) As (A) but normalized according to dCHIP 1.3. (D) As (A) but normalized according to the RMA method (Affy R package)

hybridization quality, using the probe set hybridization quality standard errors (SE) generated by dCHIP [14]. The intensity values obtained using RMA/quantile normalization are subsequently coupled to the filtered genes and used for statistical validation.

### Statistical validation of differential expression

Because microarray results are influenced by various experimental errors [4] it is important to perform replicates of the experiments in order to assess the variability of the gene expression levels in the treatment and control groups and to evaluate the statistical meaning of those variations. Statistical validation is quite important because the simple-minded fold approach, in which a gene is declared to have significantly changed if its average expression level varies by more than a constant factor, is unlikely to yield optimal results because the fold change factor can have different significance, depending on expression levels [2]. Usually, for a limited number of replicates, a parametric or non-parametric test can be carried out. When multiple hypotheses are tested, as in the case of thousands of genes present on a microarray, the probability that at least one type I error (i.e. a gene is considered differentially expressed although it is not true) is committed can increase sharply with the number of hypotheses. For these reasons, a variety of approaches have been developed to avoid this kind of error.

Significance analysis of microarrays (SAM) was developed by Tusher and co-workers [16] and is a statistical technique for finding genes showing significant differential expression in a set of microarray experiments. The input to SAM is gene expression measurements from a set of microarray experiments, as well as a response variable from each experiment. SAM measures the strength of the relationship between gene expression and the response variable and uses repeated permutations of the data to determine whether the expression of any gene is significantly related to the response. The user has to define the acceptable false

Copyright © 2003 John Wiley & Sons, Ltd.

discovery rate, and can also specify a fold change threshold.

CyberT was developed by Baldi and Long [2]; it allows the calculation of how meaningful a differential expression is using a Bayesian probabilistic framework. In particular, CyberT uses a Bayesian approach to calculate a background variance for each of the genes under analysis and it uses such values to balance experimental fluctuations within a limited number of replicates. As demonstrated by the authors [2], the Bayesian approach appears robust relative to the use of fold change alone, as large non-statistically significant fold changes are often associated with large measurement errors. In our lab, we use CyberT to validate results generated by SAM: we consider a gene differentially expressed only if it has passed the SAM test and if it is present within the top score results generated by CyberT [14].

### Conclusions

Although the methodologies described in this paper are currently the most robust tools available and are constantly updated by the developers, we have to take into account that microarray analysis is a very dynamic field and many new tools are becoming available. Therefore, it has to be accepted that, in order to grasp all of the hidden knowledge in our datasets, they must be analysed again as new appealing methodologies emerge.

### Acknowledgements

This work was partially supported by PRIN2001 2001057147 and FIRB RBAU01JTHS grants from the Italian Ministry of University and S&T Research (MIUR).

### References

1. Affymetrix. 2001. *Affymetrix Microarray Suite User Guide, version 5*. Affymetrix: Santa Clara, CA.
2. Baldi P, Long AD. 2001. A Bayesian framework for the analysis of microarray expression data: regularized *t*-test and statistical inference of gene changes *Bioinformatics* **17**: 509–519.
3. Bolstad BM, Irizarry RA, Astrand M, Speed TP. 2003. A comparison of normalization methods for high density oligonucleotide array data based on variance and bias. *Bioinformatics* **19**: 185–193.
4. Dudoit S, Yang YH, Callow MJ, Speed TP. 2002. Statistical methods for identifying differentially expressed genes in replicated cDNA microarray experiments. *Statistica Sinica* **12**: 111–139.
5. Golub TR, Slonim DK, Tamayo P, *et al*. 1999. Molecular classification of cancer: class discovery and class prediction by gene expression monitoring *Science* **286**: 531–537.
6. Hartemink DG, Jaakkola I, Young R. 2001. Maximum likelihood estimation of optimal scaling factors for expression array normalization. In *Microarrays: Optical Technologies and Informatics Proceedings of SPIE*. SPIE: Bellingham, WA; 4266.
7. Irizarry RA, Bolstad BM, Collin F, *et al*. 2003a. Summary of Affymetrix GeneChip probe level data. *Nucleic Acids Res* **31**(4): e15.
8. Irizarry RA, Hobbs B, Collin F, *et al*. 2003b. Exploration, normalization, and summaries of high density oligonucleotide array probe level data. *Biostatistics* **4**: 249–264.
9. Irizarry RA, Gautier L, Cope L. 2003c. An R package for analyses of Affymetrix oligonucleotide arrays. In *The analysis of gene expression data: methods and software*, Parmigiani G, Garrett ES, Irizarry RA, Zeger SL (eds). Springer: New York. (In press).
10. Li C, Wong WH. 2001a. Model-based analysis of oligonucleotide arrays: model validation, design issues and standard error application. *Genome Biol* **2**: 1–11.
11. Li C, Wong WH. 2001b. Model-based analysis of oligonucleotides arrays: expression index computation and outlier detection. *Proc Natl Acad Sci USA* **98**: 31–36.
12. Kim S, Dougherty ER, Chen Y, *et al*. 2000. Molecular classification of cancer: class discovery and class prediction by gene expression monitoring. *Genomics* **67**: 201–209.
13. Rocke DM, Durbin B. 2001. A model for measurement error for gene expression arrays. *J Comput Biol* **8**: 557–569.
14. Saviozzi S, Iazzetti G, Caserta E, Guffanti A, Calogero RA. 2003. Microarray data analysis and mining. In *Methods in Molecular Medicine: Molecular Diagnosis of Infectious Diseases*, Decker J, Reischl U (eds). Humana: Totowa, MA (in press).
15. Schena M, Shalon D, Davis RW, Brown PO. 1995. Quantitative monitoring of gene expression patterns with complementary DNA microarray. *Science* **270**: 467–470.
16. Tusher VG, Tibshirani R, Chu G. 2001. Significance analysis of microarrays applied to ionizing radiation response. *Proc Natl Acad Sci USA* **98**: 5116–5121.

Copyright © 2003 John Wiley & Sons, Ltd.
